# The Potential of Chitosan-Based Composites for Adsorption of Diarrheic Shellfish Toxins

**DOI:** 10.3390/toxins16040200

**Published:** 2024-04-21

**Authors:** Joana F. Leal, Patrícia S. M. Amado, João P. Lourenço, Maria L. S. Cristiano

**Affiliations:** 1 Centro de Ciências do Mar (CCMAR/CIMAR LA), Universidade do Algarve (UAlg), 8005-039 Faro, Portugal; jfleal@ualg.pt (J.F.L.); psamado@ualg.pt (P.S.M.A.); 2 Departamento de Química e Farmácia, Faculdade de Ciências e Tecnologia, Universidade do Algarve, 8005-039 Faro, Portugal; jlouren@ualg.pt; 3 Centro de Química Estrutural (CQE), Instituto de Ciências Moleculares (IMS), Instituto Superior Técnico, Universidade de Lisboa, Av. Rovisco Pais, 1049-001 Lisboa, Portugal

**Keywords:** diarrheic shellfish poisoning, marine biotoxins, okadaic acid removal, composite materials as adsorbents

## Abstract

Okadaic acid (OA) is one of the most potent marine biotoxins, causing diarrheal shellfish poisoning (DSP). The proliferation of microalgae that produce OA and its analogues is frequent, threatening human health and socioeconomic development. Several methods have been tested to remove this biotoxin from aquatic systems, yet none has proven enough efficacy to solve the problem. In this work, we synthesized and characterized low-cost composites and tested their efficacy for OA adsorption in saltwater. For the synthesis of the composites, the following starting materials were considered: chitosan of low and medium molecular weight (CH-LW and CH-MW, respectively), activated carbon (AC), and montmorillonite (MMT). Characterization by vibrational spectroscopy (FTIR), X-ray diffraction (XRD), and microscopy revealed differences in the mode of interaction of CH-LW and CH-MW with AC and MMT, suggesting that the interaction of CH-MW with MMT has mainly occurred on the surface of the clay particles and no sufficient intercalation of CH-MW into the MMT interlayers took place. Among the composites tested (CH-LW/AC, CH-MW/AC, CH-MW/AC/MMT, and CH-MW/MMT), CH-MW/MMT was the one that revealed lower OA adsorption efficiency, given the findings evidenced by the structural characterization. On the contrary, the CH-MW/AC composite revealed the highest average percentage of OA adsorption (53 ± 11%). Although preliminary, the results obtained in this work open up good perspectives for the use of this type of composite material as an adsorbent in the removal of OA from marine environments.

## 1. Introduction

**Figure 1:**
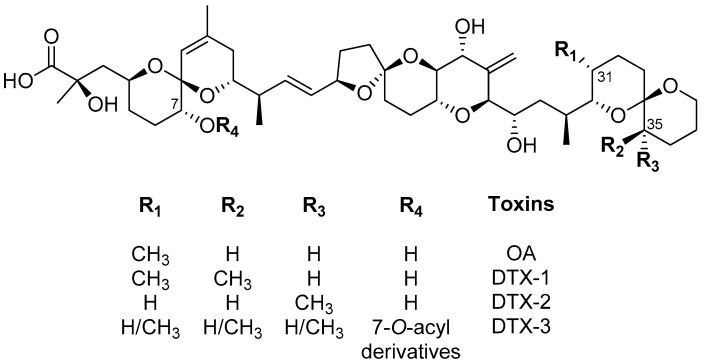
Structure representation of okadaic acid (OA) and dinophysistoxins (DTXs).

Diarrheic shellfish poisoning (DSP) poses significant threats to public health through the consumption of contaminated seafood. DSP causative toxins primarily include okadaic acid (OA) and its analogues, dinophysistoxin-1 (DTX-1) and dinophysistoxin-2 (DTX-2) (Figure 1) [1,2], that are commonly produced by the phytoplankton species. DTX toxins can undergo esterification by shellfish, and the process may involve fatty acids with carbon chains of different lengths to form 7-*O*-acyl derivatives called dinophysistoxin-3 (DTX-3) [3]. DTX-3 also includes acyl esters of OA [2]. Research on the mechanisms of toxicity of DSP toxins has shown that they inhibit the activity of serine/threonine protein phosphatases 1 (PP1) and 2A (PP2A) and are involved in many cellular functions, including cell cycle regulation, cytoskeleton rearrangement, intracellular metabolism, and gene expression [4,5]. Additionally, it was demonstrated that DSP toxins are potent tumor promoters, with genotoxic and cytotoxic effects on human and animal cells [2,4,5]. In humans, the symptoms of DSP poisoning appear between 30 minutes and a few hours after ingestion of toxic marine foods (e.g., bivalve mollusks) and are characterized by nausea, vomiting, diarrhea, and abdominal pain [6].

Although the intoxications caused by these toxins are not lethal, their presence in marine ecosystems above the legal limits—160 μg OA-eq/kg [2]—leads to the prohibition of shellfish harvesting. Such bans may last for weeks or months [7] and entail high economic losses for shellfish catchers and producers, as well as other players in the sector, such as purification centers or the processing industry. Aiming to solve this problem, different approaches to eliminating DSP toxins from shellfish have been tested, such as thermal treatments, ozonation, γ-irradiation, and feeding with non-toxic algae [8,9,10], but all were devoid of successful outcomes since these lipophilic molecules are not rapidly depurated by bivalves. The depuration rate of diarrhetic shellfish poisoning (DSP) toxins in various mussel species was investigated, considering food availability, temperature, mussel size, and lipid content. Reported toxins half-life times range widely from less than a day to 25 days [11]. Despite these efforts, the available knowledge regarding DSP toxins depuration kinetics is still scarce, and for further improvements, current methods need to be optimized and/or new technologies must be developed.

**Figure 2:**
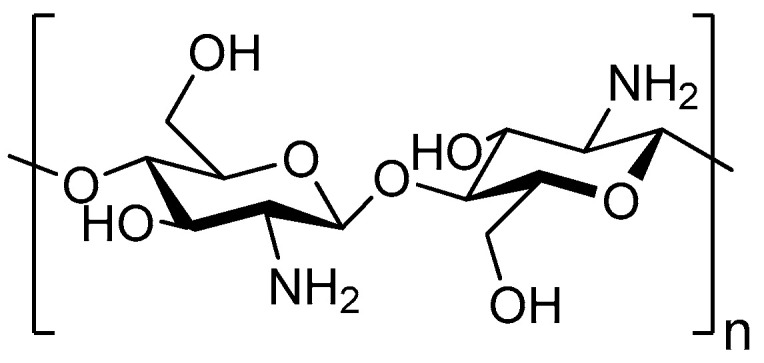
Structure representation of chitosan.

The application of adsorbents like chitosan [12], activated carbon [13], and clays [14] is one of the most promising possibilities for biotoxin elimination. Chitosan (CH) is a cationic polysaccharide derived from the *N*-deacetylation of chitin, containing the active functional groups -NH_2_ and -OH (Figure 2). Due to their hydrophilicity and a high number of hydroxyl and amino groups, CH polymers are usually employed to eliminate pollutants such as heavy metal ions, dyes, pharmaceuticals, and other toxins [15,16]. CH polymers are biodegradable, non-toxic, chemically stable, and environmentally benign [15,16], making them attractive for the detoxification of marine systems. In turn, activated carbon (AC) has shown efficacy in capturing organic micropollutants, such as pesticides [17], while clay minerals (e.g., montmorillonite, MMT) are excellent candidates for the cationic exchange reduction of hazardous metallic ions and cationic compounds in water [18]. However, the use of CH or AC as the single adsorbent has displayed various drawbacks, such as the difficulty of reusing them as well as their cost. Hence, chitosan-activated carbon (CH-AC) composites have been developed as an alternative to standard wastewater treatment processes [19,20,21,22,23]. By mixing selected adsorbents with specific and complementary adsorption properties, such as CH and AC or CH and MMT [24], it is possible to design a hybrid material that synergistically combines the single remediation capacity. Despite the versatility of these adsorbents, their interaction with DSP toxins has not been assessed yet.

In this study, we synthesized and characterized hybrid adsorbents containing AC and/or MMT powder distributed in CH biopolymers and evaluated their properties. The adsorption ability of these composites toward OA, the parent compound of DTXs (Figure 1), was investigated in saltwater, yielding a set of preliminary results that allow the selection of the best adsorbent composition. We intend for these results to be a starting point for future studies in tests with algae cultures and bivalves, where it will be possible to adjust the DSP adsorption method depending on the target (algal or bivalve cells). Okadaic acid (OA) was selected to carry out the adsorption experiments in this study given that it is the main representative and the reference analogue of the DSP toxins and possesses the greatest toxicity (toxic equivalence factor (TEF) = 1.0) [2]. 

## 2. Results and Discussion

### 2.1. Characterization of Chitosan-Based Composites

#### 2.1.1. Characterization by Vibrational Spectroscopy

**Figure 3:**
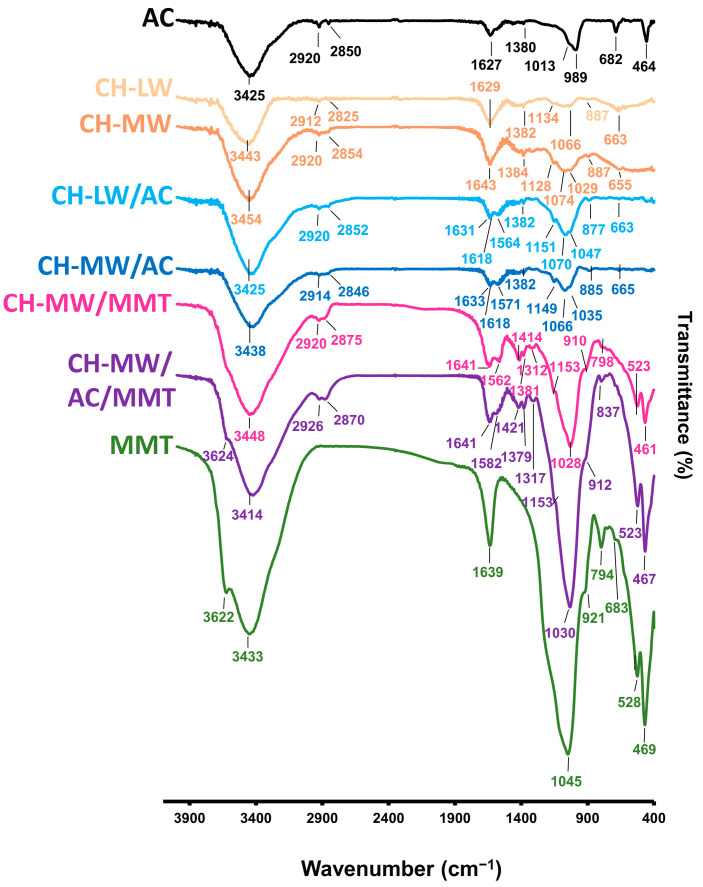
FTIR spectra of AC, CH-LW, CH-MW, MMT, CH-LW/AC, CH-MW/AC, CH-MW/MMT, and CH-MW/AC/MMT before OA adsorption (AC—activated carbon; CH-LW—low molecular weight chitosan; CH-MW—medium molecular weight chitosan; MMT—montmorillonite).

The infrared spectra of synthesized composites are presented in Figure 3. It is acknowledged that when two or more substances are mixed, chemical interactions are reflected by changes in characteristic vibrational frequencies [25].

The regions at 3000–3650 cm^−1^ in composites CH-LW and CH-MW indicate O-H stretch, which is overlapped with the N–H stretching vibration of the polysaccharide [26,27]. Absorption peaks at 2912 cm^−1^ (CH-LW) and at 2920 cm^−1^ (CH-MW), and at 2825 cm^−1^ (CH-LW) and at 2854 cm^−1^ (CH-MW) can be assigned to the C-H stretching vibration of the methyl groups (-CH_3_), which are generally observed in this region. The stretching mode of the carbonyl of the amide group is observed at 1629 cm^−1^ in the CH-LW IR spectrum and at 1643 cm^−1^ in the CH-MW IR spectrum. The IR absorption peak at 1382 cm^−1^ (CH-LW) and at 1384 cm^−1^ (CH-MW) can be assigned to the stretching vibration of the C-N bond of the amide group, as previously reported [26,28,29,30,31]. The peaks observed at 1134 and 1066 cm^−1^ (CH-LW) and 1128 and 1074 cm^−1^ (CH-MW) are attributed to the C-O stretching in chitosan [32]. The peaks at 887 cm^−1^ may be associated with the C-H bending mode [26,29,30,31]. In the FTIR spectrum of AC, there is a broad absorption observed in the OH stretching region at 3000–3600 cm^−1^, due to intermolecular interactions [33]. The peaks at 2920 cm^−1^, 2850 cm^−1^, 1013 cm^−1^, and 989 cm^−1^ may be due to aliphatic C-H stretching mode, C=C bending, and stretching of C-O and O-H deformation in carboxylic acids, –O–CH_3_ of the aldehyde group, normally found in carbonaceous material, such as activated carbon, respectively [21,34,35]. Furthermore, the absorption band in the region of about 1627 cm^−1^ is normally associated with the C=C stretching vibrations of the aromatic ring. The peak at 682 cm^−1^ may be assigned to out-of-plane bending vibration of the C-H bonds [36].

The FTIR spectra of both CH-LW/AC and CH-MW/AC show the band at 3425 cm^−1^ (CH-LW) and at 3438 cm^−1^ (CH-MW), attributed to O-H stretching vibration. Absorption peaks at 2920 cm^−1^ (CH-LW) and 2914 cm^−1^ (CH-MW) and at 2852 cm^−1^ (CH-LW) and 2846 cm^−1^ (CH-MW) are also observed, and they can be assigned to the C-H stretching vibration of -CH_3_. The absorption bands observed at 1631 cm^−1^ (CH-LW) and at 1633 cm^−1^ (CH-MW) correspond to the stretching vibration of the carbonyl, C=O, of the amide group. Slightly shifted peaks at ranges of 1618–1564 cm^−1^ (CH-LW) and 1618–1571 cm^−1^ (CH-MW) in the FTIR spectra of AC coated with CH are related to the N–H scissoring from the amine and amide groups [35]. These findings confirm that CH is successfully coated with AC. The peaks at 1382 cm^−1^ ascribed to the stretching of C-N from amide and the peaks at 877 cm^−1^ (CH-LW) and 885 cm^−1^ (CH-MW), due to the out-of-plane vibration of the N-H bond in primary and secondary amines, are also observed in both composites of CH/AC.

The spectrum of MMT showed the characteristic band at 3622 cm^−1^ due to O-H stretching and a broad peak centered on 3433 cm^−1^ attributed to interlayer and intralayer H-bonded O-H stretching. An intense and sharp IR peak at 1045 cm^−1^ can be assigned to the Si-O stretching vibration. This absorption band is red-shifted in the IR spectrum of composites with MMT, being observed at 1028 cm^−1^ for CH-MW/MMT and 1030 cm^−1^ for CH-MW/AC/MMT [37]. The characteristic peaks had shifted to lower frequencies (1562–1312 cm^−1^ for CH-MW/MMT and 1582–1317 cm^−1^ for CH-MW/AC/MMT, respectively) in chitosan blend films, which are likely due to intermolecular interactions between functional groups of CH-MW and MMT [38,39]. As expected, all the characteristic peaks of chitosan (2920/2926 cm^−1^ and 2870/2875 cm^−1^ for CH-MW/MMT and CH-MW/AC/MMT, respectively) were observed in the FTIR spectra of the chitosan blend films. The peaks at 1562 cm^−1^ (CH-MW/MMT) and 1582 cm^−1^ (CH-MW/AC/MMT) related to the N-H bending vibration, and the C-H bending absorption peak at 1414 cm^−1^ (CH-MW/MMT) and 1421 cm^−1^ (CH-MW/AC/MMT) became sharper in the films incorporated with MMT, with higher resolution in CH-MW/MMT than the CH-MW/MMT coated with AC.

#### 2.1.2. Analysis of the XRD Patterns

**Figure 4:**
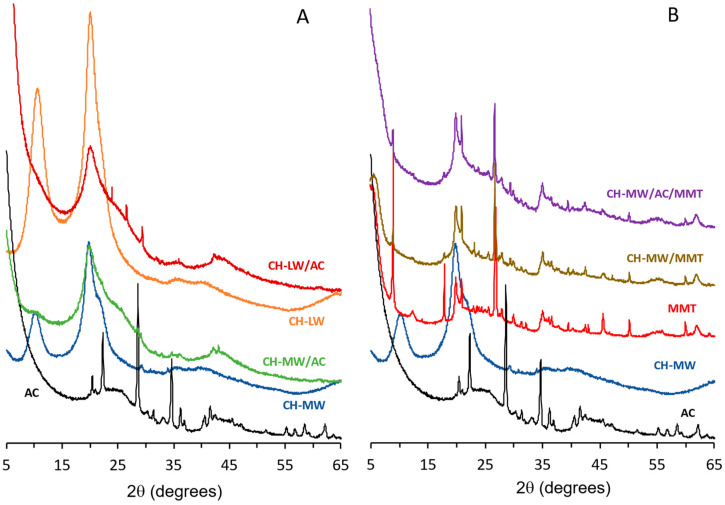
X-ray diffraction patterns before OA adsorption. Comparison between (**A**) AC, CH-MW, CH-LW, CH-LW/AC, CH-MW/AC, and (**B**) AC, CH-MW, MMT, CH-MW/MMT, and CH-MW/AC/MMT. AC—activated carbon; CH-LW—low molecular weight chitosan; CH-MW—medium molecular weight chitosan; MMT—montmorillonite.

The XRD patterns of composites are shown in Figure 4. Activated carbon (Figure 4A) shows a broad peak in the range 20–30° 2*θ*, as expected from the amorphous nature of the material. Additionally, several sharp peaks are also clearly seen in the pattern, indicating another crystalline phase that could be identified as KAlSiO_4_ (ICDD PDF-2 #01-085-1413).

The patterns of unmodified CH (Figure 4A) show diffraction peaks at ca. 10.2°, 19.7°, and 21.8° 2*θ* that can be assigned, respectively, to the (020), (110), and (120) reflexions of α-chitin [40,41,42]. The difference in peak intensity of the reflexions (020) and (120) observed for the two samples could indicate differences in the degree of deacetylation [43]. However, this indication should be considered with caution since the history of the samples is different (different manufacturers) [44].

The patterns of the composites comprising CH and AC show a significant decrease in the intensity of the peaks associated with CH materials. This decrease is stronger than expected by the dilution effect and is particularly evident for the (020) plane. This behavior is consistent with the loss of the long-range order of the polymeric chains due to the presence of activated carbon. Furthermore, for the same dilution, a higher reduction in the crystallinity of CH in the CH-LW/AC composite when compared with the sample CH-MW/AC (as indicated by the reduction of intensity and broadening of the peaks, along with the increase of the broad peak at ca. 20–30° 2*θ*), suggests a distinct interaction of activated carbon with CH-LW or CH-MW, with a more intimate mixture in the case of CH-LW due to the smaller chain length. Additionally, the pattern of the composite CH-MW/AC shows some sharp peaks of an unidentified phase (that do not correspond to those found in the AC pattern) and could be due to some impurity during the synthetic process.

The sample MMT (Figure 4B) displays several peaks that allow the identification of different crystalline phases. The broad peak at ca. 5.6° 2*θ* is characteristic of acid-treated montmorillonite [37]. In this case, a basal spacing (d001) of ca. 15.8 Å indicates a rather expanded structure. The presence of montmorillonite is also confirmed by the presence of peaks at 19.9°, 35.3°, and 61.8° 2*θ* (ICDD PDF-2 #00-003-0015) [45]. Additionally, muscovite and quartz are also crystalline phases present in the sample. The peaks at 8.9°, 19.8°, 29.9° and 35.0° 2*θ* are characteristic of muscovite, while quartz can be identified by the peaks at 20.8°, 26.6°, 50.2°, and 60.0° 2*θ* (ICDD PDF-2 #00-001-1098 and #00-005-0490, respectively). Upon inclusion of MMT in the CH-based composites, the peaks assigned to CH (10.2° and 19.7° 2*θ*) almost disappear, which may indicate the interaction between these two materials and possible intercalation of CH-MW in the MMT interlayers [46,47]. However, if we compare the XRD pattern of MMT with that of the composite CH-MW/MMT, no deviations in the positions of the main peaks (towards smaller angles) are observed. According to Bragg’s law [48], the deviation of peaks to lower angles would be associated with a wider distance (higher d-spacing) between the MMT interlayers. As the position of peaks remained practically constant when comparing the pattern of CH-MW/MMT with that of MMT, namely the peak at 2*θ* = 5.6° that is associated with the interlayer basal planes, it could mean that under these experimental conditions, the interaction of CH-MW with MMT has mainly occurred on the surface of the clay particles and no sufficient intercalation of CH-MW into the interlayers took place. This behavior is in keeping with that already observed by other authors [37]. The pattern of the composite containing AC (CH-MW/AC/MMT) still shows the presence of the peaks associated with the layers themselves (which do not depend on the basal spacing) [49], showing that the structural integrity of the layers is retained. Nevertheless, some changes in the relative peak intensities are observed. The peak at 5.6° (associated with the basal spacing of MMT) seems to vanish, and the peak at 8.9° appears significantly less intense. Although a reduction in peak intensity would be expected due to the dilution effect, these observations, particularly those concerning the peaks above, also suggest that the layered materials could have suffered some exfoliation process [50,51]. 

#### 2.1.3. Microscopy Analysis 

**Figure 5:**
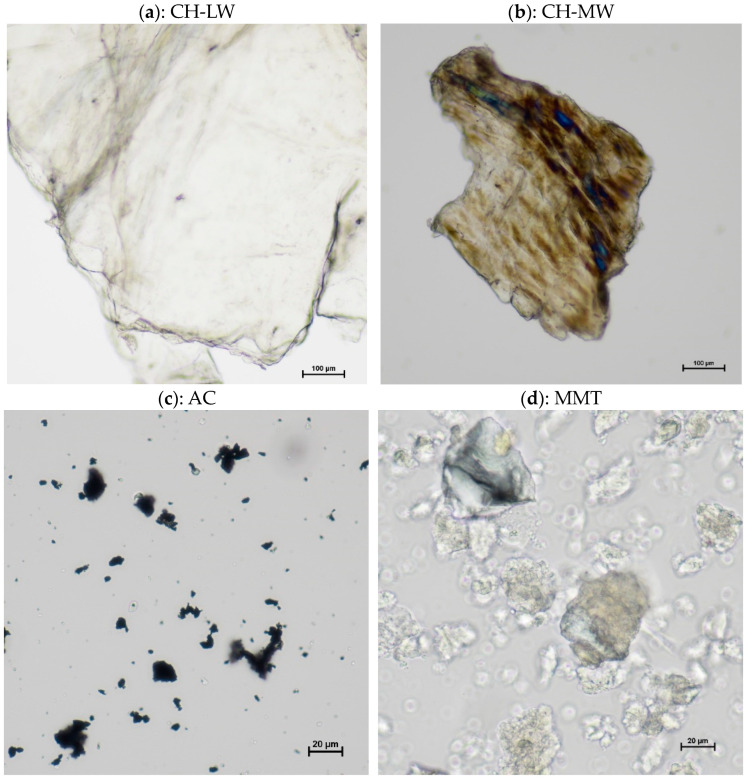
Microscopy images of CH-LW (**a**) and CH-MW (**b**), both at 10× magnification (the tick mark is equal to 100 μm), and AC (**c**) and MMT (**d**), both at 40× magnification (the tick mark is equal to 20 μm). AC—activated carbon; CH-LW—low molecular weight chitosan; CH-MW—medium molecular weight chitosan; MMT—montmorillonite.

**Figure 6:**
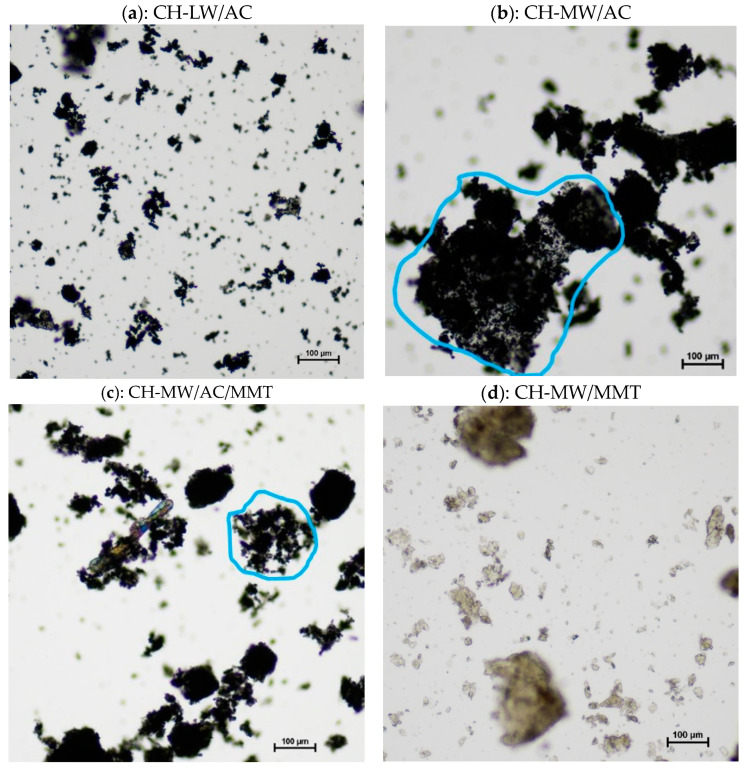
Microscopy images of CH-LW/AC (**a**), CH-MW/AC (**b**), CH-MW/AC/MMT (**c**), and CH-MW/MMT (**d**), all at 10× magnification (the tick mark is equal to 100 μm). AC—activated carbon; CH-LW—low molecular weight chitosan; CH-MW—medium molecular weight chitosan; MMT—montmorillonite. The areas highlighted by the blue line (images **b**,**c**) are examples of the interaction/encapsulation of AC particles in the CH-MW polymeric “sheet”.

Optical microscopy was used to evaluate the morphology of the materials as well as their interaction as composites. Some of the images obtained are shown in Figure 5 and Figure 6.

The sheet appearance that characterizes CH is in accordance with its linear polyamine structure. The differences observed between images of CH-LW (Figure 5a) and CH-MW (Figure 5b) are attributed to the level of entanglement, which is enhanced by the increase in molecular weight [17,52]. Although CH-MW structures appear smaller than CH-LW structures, in fact, they are just more tangled up on themselves, conveying this idea of smaller size. Figure 5c shows dispersed amorphous carbonaceous materials characteristic of AC [53], while Figure 5d presents the well-defined crystalline structure of MMT (best seen in the foreground), which consists of the overlap of several silicate layers [54].

One of the great functional advantages of using CH as a base compound is its encapsulation potential. Given the individual structures of AC and CH, it is expected that the AC will be encapsulated in the CH polymeric framework [55]. This is corroborated by Figure 6b,c, where the interaction/encapsulation of AC particles in the CH-MW polymeric “sheet” is clearly observed (examples highlighted by blue outline), evidencing the successful synthesis of the CH-MW/AC composites. Interestingly, the interaction between CH-LW and AC is not evident, and the image obtained is very similar to that of AC alone (Figure 6a vs. Figure 5c). In addition to the differences in molecular weight between the different types of CH tested (low vs. medium molecular weight), their deacetylation degree (DD) differs slightly, with CH-LW having a slightly higher DD (a higher percentage of amino groups in the molecular chain) than CH-MW. Some authors [56] have reported that molecular weight influences the surface properties, as well as the content of functional groups and the structure of the molecular chains, in the case of CH with similar DD. Both structural parameters (DD and molecular weight) affect the interaction of CH with other compounds, which could justify the different behavior observed in Figure 6a,b. In Figure 6d, the CH-MW “sheets” (higher structures) and MMT crystals (smaller structures) are distinguished separately, suggesting a weak or even no interaction between both materials. Under a simplistic view, the positive charge associated with CH and the negative charge associated with MMT would favor an adsorption mechanism based on electrostatic attraction. However, adsorption may also be influenced by other parameters, namely solubility, pH, and the concentration of other electrolytes in the medium [57]. As in the adsorption studies, the microscopy images of the compounds were obtained in saline solution, which implies the presence of other electrolytes in high concentration in the medium as well as a slightly basic pH value. For higher pH values, the amino groups are deprotonated, meaning that the CH assumes a more neutral character and the adhesion to MMT could be lower. Furthermore, neutral amino groups interact with positive counterions, such as Na^+^, which will also affect the interaction mode of CH with MMT [58].

### 2.2. Adsorption Studies—OA Quantification

**Figure 7:**
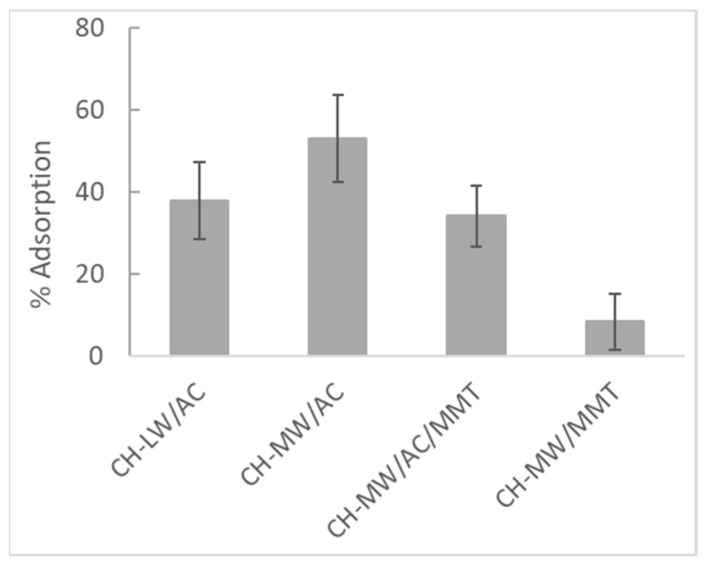
Adsorption percentages of OA (100 μg/L) in saline solution (33 ppt) after 30 min of contact with different chitosan (CH)-based composites with magnetic stirring (120 rpm). The concentration of each composite used was equal to 1 g/L. AC—activated carbon; CH-LW—low molecular weight chitosan; CH-MW—medium molecular weight chitosan; MMT—montmorillonite. Vertical error bars correspond to standard deviations (*n* = 3).

The ability of CH-based composites to remove, by adsorption, one of the most frequent and dangerous biotoxins causing DSP–okadaic acid (OA)—was evaluated, and the process was followed using high-performance liquid chromatography with fluorescence detection (HPLC-FLD). Figure 7 shows the results obtained with the following combinations: (i) CH-LW/AC, (ii) CH-MW/AC, (iii) CH-MW/AC/MMT, and (iv) CH-MW/MMT.

It is known that adsorption efficiency is influenced by several parameters, including the size of the adsorbent particles [59]. In this work, it was not possible to use a mesh with a specific porosity after grinding the composites. This may have contributed to a lesser homogeneity in the size of the adsorbent particles and, consequently, to a higher standard deviation between replicates. Even so, the results obtained allow us to draw some conclusions and relate them to the characteristics of the tested composites. Among all CH-based composites, it is evident that the CH-MW/MMT combination is the least efficient (lowest percentage of adsorption, 8.5 ± 6.9%) and most unstable (highest relative standard deviation, %RSD = 81). Some authors have demonstrated the synergic effect resulting from the combination of CH and MMT on the adsorption of anionic dyes, but their studies were carried out at pH = 4.4 [50]. At these acidic conditions, amino groups of CH are positively charged, prevailing electrostatic interactions, namely attractive forces, between the CH-MW/MMT composite and an anionic compound. On the contrary, in our work, the adsorption experiments were performed at the typical pH of seawater, meaning an overall lower positive or negative charge of the CH-MW/MMT composite. In these conditions, repulsive forces between this composite and OA could prevail, justifying the lower OA adsorption. Also, the lower efficiency of this combination (CH-MW/MMT) could be corroborated by the findings observed in the XRD patterns (Figure 4B) and in the microscopy analysis (Figure 6d), which indicated poor interactions between CH-MW and MMT. This means that, under the experimental conditions used, namely in a saline solution with a higher pH value, the synergistic effect that could arise from the combination of these two adsorbents was not observed. 

Regarding the remaining three compositions tested for OA adsorption, although the differences between them are not statistically significant, the average percentage of OA removal in the presence of CH-MW/AC is the highest (53 ± 11%). Observing the results obtained with CH-MW/AC/MMT, the average percentage of OA adsorption (34 ± 7%) tends to be lower than with CH-MW/AC and is clearly higher than that obtained with CH-MW/MMT. Under the conditions tested, this suggests that the adsorption capacity of CH-MW/AC is somewhat inhibited by the presence of MMT, probably for the reasons previously mentioned, counterbalancing in a certain way the balance of attractive and repulsive forces between the composites and OA. In turn, the apparent lower OA adsorption capacity of CH-LW/AC (38 ± 9%) compared to CH-MW/AC may be associated with its higher DD (higher percentage of amino groups in neutral form under the conditions tested). Also, the findings revealed in the characterization of these composites of different molecular weights may corroborate their apparent different capacity for interaction and adsorption of OA, as previously discussed. 

Although preliminary, the results obtained in this work are promising, especially those obtained using CH-based composites with AC. In addition to the easy access to these compounds individually and the simplicity of the synthesis of the respective composites, these are relatively cheap adsorbents whose properties may be optimized, aiming to increase the adsorption efficiency of OA and its derivatives (DTXs). Even so, our results reveal better adsorption efficiency than that obtained from other studies reported in the literature, for example, those using polymeric resins [60] or plastic fragments [61] as adsorbents. In the last case, 10 μg/L of OA was spiked into seawater containing fragments of polyethylene terephthalate (PET), polypropylene (PP), expanded polystyrene (EPS), and non-expanded polystyrene (PS) for 96 h [61]. The authors reported removal percentages between 30 and 83%, but after 48 h of contact and using 4 g/L of adsorbent, which are values much higher than those tested in this work (30 min and 1 g/L). We are thus convinced of the great potential of our composites in the adsorption of OA.

## 3. Conclusions

In this work, different CH-based composites were synthesized, characterized, and tested for the adsorption of OA in an aqueous saline solution. The findings revealed by the different characterization techniques (FTIR, XRD, and microscopy) confirm that CH is successfully coated on AC and that AC interacts with CH-LW and CH-MW. The results also suggest that the interaction of CH-MW with MMT has mainly occurred on the surface of the clay particles, and no sufficient intercalation of CH-MW into the interlayers occurred. This finding, combined with the charge balance between the composite and OA in a saline environment, may justify the lower adsorption capacity observed with the CH-MW/MMT composite. On the contrary, the average percentage of OA removal is the highest in the presence of CH-MW/AC (53 ± 11% in 30 min). Additionally, the remaining average percentages of OA removal seem to indicate that the presence of MMT inhibits the adsorption capacity of CH-MW/AC. The preliminary tests presented in our study provide valuable contributions to the choice of materials and conditions to be considered in future in vivo studies, with a view to approximating to the real conditions for applying the methodology.

## 4. Materials and Methods

### 4.1. Chemicals

For the synthesis of CH-based composites, chitosan of low (CH-LW) and medium (CH-MW) molecular weight from TCI (Tokyo, Japan) and Sigma-Aldrich (Darmstadt, Germany), respectively, was used. Their deacetylation degrees are ≥80.0% for CH-LW and ≥75% for CH-MW. The unmodified montmorillonite K10 (MMT) was supplied by Thermo Scientific (Waltham, MA, USA), while activated charcoal (or carbon), granulated 1–4 mm, was purchased from Labkem (Dublin, Ireland). Glacial acetic acid (≥99.8%) from Carlo Erba Reagents (Cornaredo, Italy) and NaOH (pellets, p.a.) from Chem-Lab NV (Zedelgem, Belgium) were used in the preparation of the solvents employed during the synthesis. 

Certified reference materials of okadaic acid sodium salt (CRM-02-OA), supplied by CIFGA S.A. (Lugo, Spain), were used for the adsorption studies and subsequent quantification.

### 4.2. Synthesis of CH-Based Composites with AC or/and MMT

**Figure 8:**
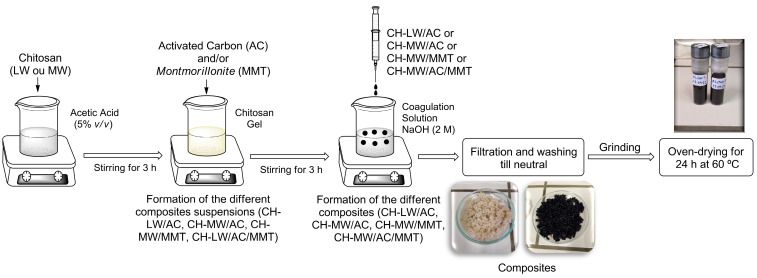
Representation of the procedure followed for the preparation of CH-LW/AC, CH-MW/AC, CH-MW/MMT, and CH-MW/AC/MMT composites. Legend: AC—activated carbon; CH-LW—low molecular weight chitosan; CH-LW—medium molecular weight chitosan; MMT—montmorillonite.

The preparation of CH-based composites with AC (CH-LW/AC or CH-MW/AC) was performed as described by Elwakeel et al. [62], with slight modifications. After mixing AC with CH-LW or CH-MW, the solution was added dropwise to 200 mL of NaOH 2 M under vigorous stirring (1500 rpm) at room temperature until a black precipitate was formed (Figure 8). CH-LW and CH-MW were considered for the mixtures to understand if their different molecular characteristics have any influence on the adsorption. 

The preparation of CH-based composites with MMT (CH-MW/MMT) and with AC and MMT (CH-MW/AC/MMT) was adapted from the report of Bouyahmed et al. [46], with slight modifications. The CH-MW powder was dissolved in acetic acid at 0.5 M to obtain a former gel mass concentration of 3%. The MMT and AC powders with a mass ratio of 3% were dispersed in the same volume of acetic acid (0.5 M) and left to stir (1500 rpm) at room temperature for 3 h, or until a homogenous gel was reached. MMT or AC/MMT were added step by step in the mixture to obtain the desired concentrations (1:1 or 1:1:1), and the dispersion mixture was stirred for 24 h. To 200 mL of NaOH 2 M, the CH-MW/MMT or CH-MW/AC/MMT mixture was added, drop-by-drop, under vigorous stirring (1500 rpm), at room temperature, until a brown or black precipitate was formed (Figure 8). 

In both procedures, each precipitate was then filtered using a Büchner funnel and PVDF membrane filter paper (43–48 µm) (Filter-Lab, Barcelona, Spain). The CH-LW/AC, CH-MW/AC, CH-MW/MMT, or CH-MW/AC/MMT beads were washed with distilled water until neutral pH and then oven-dried at 60 ºC for 12 h. The composites were then ground into a fine powder using a mortar and pestle and kept in glass vials shielded from light at room temperature.

### 4.3. Chitosan-Based Composites Characterization

The synthesized composites, as well as the starting materials, were characterized by Fourier-transform infrared spectroscopy (FTIR), X-ray diffraction (XRD), and microscopy. The FTIR spectra (transmission) were measured in the range of 4000–400 cm^−1^, at a resolution of 4 cm^−1^, using a Bruker Tensor27 spectrometer (Billerica, MA, USA). Samples were compressed onto discs and analyzed against KBr. 

Powder X-ray diffractograms were obtained from a PANalytical X’Pert Pro (Almelo, Netherlands) diffractometer operating at a voltage of 50 kV and a current of 30 mA, using Cu-Kα radiation filtered by Ni. The patterns were recorded in reflexion mode using an X’Celerator detector (Almelo, Netherlands) with a step size of 0.03° and a time per step of 350 s. 

The microscopy images were acquired with a high-resolution color camera (Nikon Digital Sight 10, Tokyo, Japan; Nikon Europe B.V., Amstelveen, The Netherlands) connected to a Nikon upright microscope Eclipse Ni with Plan Fluor Ph Objectives (Nikon Europe B.V., Amstelveen, The Netherlands) (10× 0.3 WD 16 MM and 40× 0.75 WD 0.66 MM). Samples were illuminated with a LED light source for brightfield and differential interference contrast (DIC) microscopy. Before imaging, powder samples were dispersed in saline solution and then mounted on borosilicate glass and coverslip slides (VWR thickness #1.5).

### 4.4. Adsorption Studies

Saline solutions of OA 100 μg/L (1.20 × 10^−1^ μmol/L) were used to carry out the adsorption experiments. For that, an aliquot of okadaic acid sodium salt in methanolic solution (certified reference material, CRM-02-OA, from CIFGA Laboratory S.A., Spain) was withdrawn and diluted in artificial seawater. This solution was previously prepared using sea salts (Classic Sea Salt, Tropic Marin, Wartenberg, Germany) to a final concentration of 33 ppt. Adsorption studies were carried out at room temperature for 30 min with magnetic stirring (120 rpm). The following composite combinations were tested: (i) CH-LW/AC, (ii) CH-MW/AC, (iii) CH-MW/MMT, and (iv) CH-MW/AC/MMT. The composite concentration used was 1 g/L. At least three independent replicates were made, with respective controls (OA saline solution without any composite) and blanks (saline solution with composites, without OA). Then, the magnets were removed, and the samples were filtered into glass vials using syringes and 0.45 µm PVDF syringe filters (Labfil) for subsequent treatment and analysis by high-performance liquid chromatography with fluorescence detection (HPLC-FLD).

### 4.5. Determination of DSP Toxins

At the time of carrying out this study, it was not possible to use the LC-MS/MS technique to quantify toxins due to constraints related to equipment availability. Although older, the protocol adopted in this study (pre-column HPLC-FLD with BAP derivatization) was validated and used by several authors previously. The protocols followed for the determination of OA were based on previous studies [63,64,65], with slight changes detailed below. 

#### 4.5.1. Extraction and Derivatization

After adsorption studies, all aqueous samples were subjected to a liquid–liquid extraction procedure with the aim of transferring OA from the aqueous phase to the organic phase. For that, 1.5 mL of ethyl acetate p.a. (≥99.5%, Honeywell Riedel-de Haën^TM^, Seelze, Germany) was added to 1.4 mL of each aqueous sample, vortexed for 1 min, and centrifuged at 1500× *g* for 1 min. Then, the upper ethyl acetate layer was transferred to a graduated test tube using a Pasteur pipette. These steps were repeated three times. The three organic fractions from each sample were combined in the same graduated tube, and the final volume was adjusted to 4.5 mL. The same procedure was applied to controls and blanks. In order to evaluate possible losses during the OA extraction process from the aqueous saline solution, two OA solutions (100 μg/L) were prepared. Both were subjected to BAP derivatization and SPE clean-up steps, but only one of them was subjected to the extraction process. This experiment was repeated independently three times, with respective analysis replicates (*n* = 3). The results obtained indicate losses of 23.5 ± 8.8% during the extraction procedure.

For derivatization, 1000 μL of each extract obtained previously was evaporated to dryness with N_2_. Thereafter, 80 μL of 2-bromo-1-(pyren-1-yl)ethanone (BAP, 0.2% *w*/*v*) and 20 μL of *N*-ethyldiisopropylamine (DIPA, 10%) were added to each vial and left to react in a sonicator, protected from light, for 20 min. Then, the samples were heated in a water bath at 75 °C for 30 min and finally evaporated with N_2_. To prepare the diluted solutions of BAP 0.2% *w*/*v* and DIPA 10%, both in acetonitrile (≥99.9%, Honeywell, Charlotte, North Carolina, United States), BAP 95% and DIPA 99% were purchased from Biosynth Ltd. (Staad, Switzerland) and Thermo Scientific Chemicals (Waltham, MA, USA), respectively. 

#### 4.5.2. Solid-Phase Extraction (SPE)

Silica cartridges 650 mg/6 mL (Teknokroma, Barcelona, Spain) were first conditioned with 3 mL of hexane–dichloromethane (1:1, *v*/*v*). Then, 3 portions of 500 μL of hexane–dichloromethane (1:1, *v*/*v*) were added to the vials containing the samples, vortexed, and transferred to the SPE cartridges. After this, two sequential washes were carried out: the first with 5 mL of hexane–dichloromethane (1:1, *v*/*v*) and the second with 5 mL of dichloromethane–ethyl acetate (8:2, *v*/*v*). Finally, elution was achieved with 4 mL of dichloromethane–methanol (9:1, *v*/*v*). The eluted samples were filtered using syringes and 0.22 μm PVDF syringe filters and subsequently evaporated with N_2_. Finally, the analyte was redissolved in 100 μL of acetonitrile and vortexed. For these procedures, dichloromethane stabilized with amylene (HPLC grade), hexane (≥95%), ethyl acetate p.a. (≥99.5%), and methanol (≥99.9%, LC-MS Chromasolv^TM^, Seelze, Germany) was acquired from Honeywell Riedel-de Haën^TM^ (Seelze, Germany).

#### 4.5.3. HPLC-FLD Analysis

**Figure 9:**
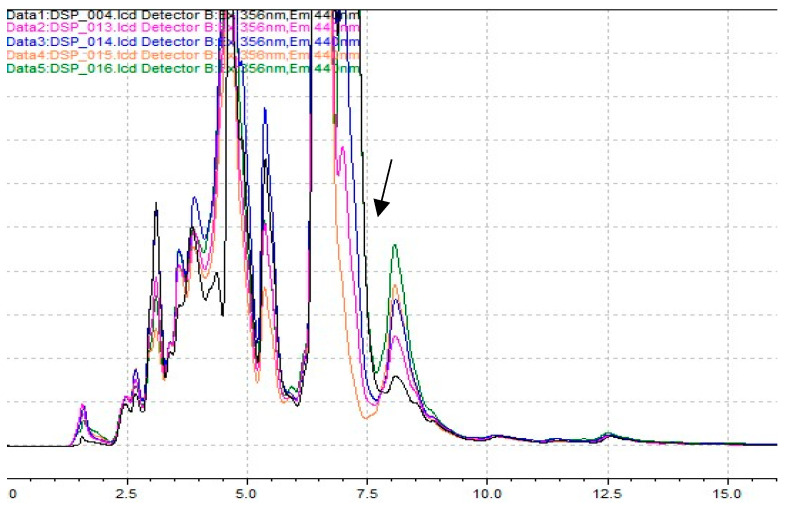
Representative chromatograms of OA (identified by the arrow), at different levels of concentration were obtained using pre-column HPLC-FLD with BAP derivatization.

An HPLC-FLD (Prominence-i LC-2030C Plus) from Shimadzu and a reversed-phase C18 column (Mediterranea Sea18, Teknokroma, Barcelona, Spain), 25 × 0.46 cm (5 μm), preceded by an ultraguardTM column (Sea18 10 × 3.2 mm, Teknokroma), were used to carry out the analyses. Details about the equipment are described in a previous work [66]. Acetonitrile and deionized water (85:15, *v*/*v*), both filtered using PVDF membrane filters (0.22 μm, 47 mm, Teknokroma), were used as the mobile phase in isocratic mode. The flow rate was 1.1 mL/min, and the injection volume was 20 μL. Fluorescence detection was achieved at 356 nm (excitation) and 440 nm (emission). Representative chromatograms are shown in Figure 9.

For quantification of OA, calibration solutions (30–120 μg/L) were prepared from intermediate solutions of OA (0.2 μg/mL) in methanol, which were previously prepared from the certified reference materials CRM-02-OA (okadaic acid sodium salt). The calibration standards were also subjected to derivatization steps to allow their analysis. The quantification of OA was performed by interpolating the data into the corresponding calibration curve. At least 3 injections of each calibration standard were made (intra-assay precision, RSD ≤ 5%). The signal-to-noise ratio (S/N) was used to estimate the limit of detection, LOD (S/N = 3), and the limit of quantification, LOQ (S/N = 10). For the different calibration curves, the LOD and LOQ calculated ranged between 4.8 and 8.8 μg/L and between 14.8 and 19.4 μg/L, respectively. All determination coefficients (R^2^) were above 0.988.

The adsorption percentages of OA from the saline solution were calculated according to Equation (1), where *C_f_* is the concentration of OA after 30 min in contact with the composites and *C_c_* is the concentration of OA after 30 min, but without any composites (control). In this way, it is guaranteed that the decrease in OA concentration is due to adsorption by the composites.

(1) $$\%\;Adsorption=\left[1-\left(\frac{C_{f}}{C_{c}}\right)\right]\times 100$$

## Data Availability

Data are contained within the article.
